# Reducing the time-lag between onset of chest pain and seeking professional medical help: a theory-based review

**DOI:** 10.1186/1471-2288-13-15

**Published:** 2013-02-06

**Authors:** Susan K Baxter, Peter Allmark

**Affiliations:** 1 Section of Public Health, School of Health and Related Research, University of Sheffield, Regent Court, 30 Regent Street, Sheffield S1 4DA, UK; 2 Health and Social Care Research Centre, Sheffield Hallam University, 32 Collegiate Crescent, Sheffield, S10 2BP, UK

**Keywords:** Systematic review, Theory-based review, Cardiac delay, Chest pain

## Abstract

**Background:**

Research suggests that there are a number of factors which can be associated with delay in a patient seeking professional help following chest pain, including demographic and social factors. These factors may have an adverse impact on the efficacy of interventions which to date have had limited success in improving patient action times. Theory-based methods of review are becoming increasingly recognised as important additions to conventional systematic review methods. They can be useful to gain additional insights into the characteristics of effective interventions by uncovering complex underlying mechanisms.

**Methods:**

This paper describes the further analysis of research papers identified in a conventional systematic review of published evidence. The aim of this work was to investigate the theoretical frameworks underpinning studies exploring the issue of why people having a heart attack delay seeking professional medical help. The study used standard review methods to identify papers meeting the inclusion criterion, and carried out a synthesis of data relating to theoretical underpinnings.

**Results:**

Thirty six papers from the 53 in the original systematic review referred to a particular theoretical perspective, or contained data which related to theoretical assumptions. The most frequently mentioned theory was the self-regulatory model of illness behaviour. Papers reported the potential significance of aspects of this model including different coping mechanisms, strategies of denial and varying models of treatment seeking. Studies also drew attention to the potential role of belief systems, applied elements of attachment theory, and referred to models of maintaining integrity, ways of knowing, and the influence of gender.

**Conclusions:**

The review highlights the need to examine an individual’s subjective experience of and response to health threats, and confirms the gap between knowledge and changed behaviour. Interventions face key challenges if they are to influence patient perceptions regarding seriousness of symptoms; varying processes of coping; and obstacles created by patient perceptions of their role and responsibilities. A theoretical approach to review of these papers provides additional insight into the assumptions underpinning interventions, and illuminates factors which may impact on their efficacy. The method thus offers a useful supplement to conventional systematic review methods.

## Background

Previous systematic reviews have suggested that there are a number of factors which can be associated with patient delay in seeking professional help following the onset of chest pain, including demographics (such as gender, race and age), and social aspects such as neighbourhood income [1]. Authors have hypothesised that these factors have an adverse effect on the success of interventions such as publicity campaigns, which to date have had limited success in improving patient action times [2,3].

A systematic review carried out by the second author concurred with previous reviews in identifying evidence of a range of suggested associations with patient delay [4]. The elements associated with delay were categorised as socio-demographic, clinical, emotional, and cognitive. The review concluded that further evidence is needed to explain the role of these factors and differences in delay between people having similar symptoms, if an effective intervention is to be developed.

Conventional systematic review approaches have been criticised by some authors as leading to disappointingly inconclusive findings regarding the success or failure of interventions, due to their lack of examination of contextual factors and perception of those taking part in programmes as being passive recipients [5]. The lack of appreciation of factors of process or full examination of the fidelity of an intervention has also been highlighted [6].

In response to some of these criticisms, theory-based methods of review are becoming increasingly recognised as important additions to conventional systematic review methods. Theory-based methods include a range of approaches such as Realistic Evaluation, Theories of Change and logic models. While the precise methods have differences, they share an aim of providing additional explanations on complexity, causal pathways and the success or failure of interventions [7]. These approaches focus on assessing the validity of the theory on which an intervention is built and are concerned with opening up the “black box” of interventions and outcomes to uncover underlying mechanisms [8]. It is argued that these developing methods are important as, without having a clear understanding of the assumptions underlying an intervention and how it is supposed to work, evaluators cannot ascertain whether it did work and why it did or did not achieve the intended benefits [9].

This paper describes the supplementary analysis of research studies identified in a previous systematic review of published evidence [4]. The aim of this work was to adopt a theory-based review approach to further illuminate the question of why people having a heart attack delay seeking professional medical help, and how effective interventions may be best designed to address this.

## Methods

The study further examined papers identified in a prior review which had used established systematic review methods (encompassing identification of papers by developing a search strategy, electronic database searching and sifting, reference list checking, citation searching, quality appraisal, data extraction and narrative synthesis). The work reported here was carried out subsequent to this conventional review by taking the pool of papers identified and carrying out further selection, extraction and synthesis based on theoretical underpinnings.

### Search strategy

Studies considered for this work had been identified in a previous review of evidence. This review had searched for papers published from 2006 to February 2011. The 2006 cut-off was selected for that work as it is the date that percutaneous coronary intervention became the widely used treatment for myocardial infarction. The work reported here was carried out with the set of included papers from this prior review rather than carrying out an independent search. It intended to investigate whether further insights could be obtained by trialling an alternative method of analysis. The search encompassed electronic database searching, reference list checking of included papers and checking of relevant reviews for additional citations of potential relevance. The databases searched were: Medline; CINAHL plus; PsycINFO; ASSIA; Web of Science; Scopus; Science Direct; DARE; Cochrane Library; and Google Scholar. Search terms were clustered around the themes of myocardial infarction, seeking help and delay. In each theme synonyms and related terms were used. The full search strategy is available from the authors.

### Study selection

Studies published in English in a peer-reviewed journal of any research design were eligible for inclusion. Research designs could thus encompass those reporting interventions, those describing associations, qualitative data relating to views and perceptions, and systematic reviews. Following a process of sifting the retrieved citations, a total of 53 papers were identified as of relevance from a database of 118 for the first conventional review. These 53 papers formed the pool of studies considered for selection in this further analysis using a theory-based review approach.

### Inclusion criteria

**Figure 1:**
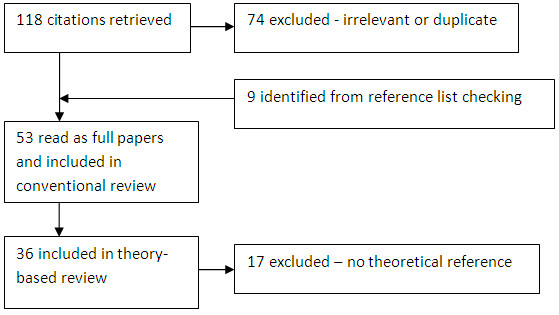
Flow chart illustrating the process of inclusion and exclusion.

The inclusion criterion for the work reported here was that the paper included data relating to a theoretical framework or referred to a theoretical mechanism. The definition of a theoretical mechanism or underpinning used was that the paper contained reference to a model or framework which the authors referred to as influencing their study design or informing the findings. Studies which were excluded contained no reference to a mechanism or theory being influential on study design, or made no reference to theories or frameworks when reporting or discussing the study findings. Potential articles for inclusion were screened by two members of the team. Figure 1 illustrates the process of inclusion/exclusion.

### Quality assessment

A wide range of critical appraisal tools are available, with a review in 2004 identifying 121 different checklists [10]. There is however no “gold standard” critical appraisal tool for any study design, nor is there any widely accepted generic tool that can be applied equally well across study types [10]. Due to the heterogeneity of study design included we created a star rating system based on the study design and quality of data analysis. By incorporating both design and analysis criteria in one tool we were able to integrate the full range of study types and avoid privileging solely by design. Four star papers had a controlled design and high quality analysis. Three star papers had a large (over 250) sample and included high quality statistical or qualitative analysis, or were high quality systematic reviews. Two star papers had smaller samples with high quality analysis, or were large samples with less rigorous analysis, or were systematic reviews with weaker analysis. One star papers had small samples with poorer quality analysis or were general reviews of the literature.

### Data extraction

The extraction process was in line with standard methods by systematically using a form to extract data from included papers. The extraction table contained columns detailing study/author/date, detail of study design, the study population, detail of the intervention if appropriate, and outcomes reported. In addition the extraction table had a column focussing on coding data describing theoretical perspectives underpinning the studies, or data which could be conceived as relating to theoretical underpinnings. Extractions were carried out by the first author and checked by a second member of the team.

### Data synthesis

Data were of both quantitative and qualitative types, thus a narrative synthesis approach was adopted [11]. The work sought to adopt a general theoretical perspective for examining the data, rather than drawing on a particular method such as realist synthesis [12]. It aimed to identify and synthesise theories, assumptions and hypothesised mechanisms within the set of papers. The extraction table was examined to identify and group studies which referred to a particular theoretical underpinning. Each sub-group of studies was then compared and contrasted exploring the study designs, populations, and outcomes to develop a synthesis of the characteristics of each sub-group of included papers.

## Results

Thirty six papers (relating to 29 studies) were identified which referred to a particular theoretical perspective or contained data which could be related to theoretical assumptions. Five papers reported randomised controlled trial data (RCT) relating to a pilot and a single study, 14 reported results from questionnaire tools and other scaled data, 12 were qualitative studies, four were secondary data analyses and one was a systematic review. The majority of the studies were rated as ** for quality, with the RCTs and three large-scale surveys achieving the highest grades. Many of the studies using questionnaires reported that the tools were designed specifically for the study, rather than utilising standardised measures. Studies also tended to rely on self-reported recollection of delay time. See Table 1 for a summary of the included studies. The seventeen studies which were excluded contained no reference to any theory or framework which acted as an underpinning rationale for the design of the study or which was used to inform understanding of the findings. The group consisted of seven analyses of patient data, five studies using patient questionnaires, two cohort studies, one RCT, one using interview and questionnaire, and one interview study. Eight of the excluded papers were graded as three stars, six as two stars and three as one star.

**Table 1:** Summary of the included studies

**Authors/date**	**Study design**	**Population**	**Outcomes**	**Quality grade**
Albarran et al., 2007	Interview	12 women with coronary heart disease (CHD)	Description of symptoms	**
Banks & Dracup K, 2007	Questionnaire and interview	61 patients with acute myocardial infarction (AMI), half male/female	Response to Symptoms Questionnaire, hours between symptoms and hospital admission	**
Buckley et al., 2007	RCT	200 people with history of CHD, mixed gender	Modified Response Questionnaire	****
Bunde & Martin R, 2006	Questionnaire and interview	433 post-AMI patients, 71% male	Neuroticism questionnaire, depression questionnaire, reported behaviour	**
Dracup et al., 2009	RCT	3522 patients with CHD	Time to presentation, Acute Cardiac Symptoms (ACS) Response Index	****
Dracup et al., 2008	Questionnaire	3522 patients with CHD	ACS Response Index Knowledge Scale	**
Fukuoka Y, 2007	Interview	1059 patients with AMI, 745 male	Description of symptom attribution	**
Fox-Wasylyshyn et al., 2010	Questionnaire and interview	135 recently diagnosed AMI patients	Symptom Congruence Scale, Coping with Heart Attack Symptoms Scale, report of delay, views regarding attribution of symptoms	**
Fox-Wasylyshyn, 2007	Secondary analysis of patient data	109 patients with previous AMI and 26 with no history of AMI	Likert scales with items relating to coping strategies	**
Galdas et al., 2007	Interview	56 males diagnosis of AMI	Views, perceptions of behaviour and common understandings	**
Gallagher et al., 2010	Interview	10 women post AMI	Reported experiences	**
Harralson, 2007	Interview and questionnaires	65 female patients with CHD	Reported barriers to delay, self-rating of health and support, depression inventory	**
Hwang et al., 2006	Secondary analysis of questionnaire data	239 patients with AMI	Myocardial Infarction Symptoms Profile, Representation of Heart Attack Symptoms questionnaire, delay time	**
Henriksson et al., 2007	Focus group	13 AMI patients and 14 relatives mixed gender	Recollection of thoughts and behaviours	**
Herning et al., 2010	Interview	14 female patients with STEMI	Recollection of thoughts and behaviours	**
Higginson, 2008	Interview	25 post-MI women	Recollection of thought processes and coping strategies	**
Kaur et al., 2006	Interview	27 AMI patients, 59% male	Recollection of thoughts and behaviours	**
Khan et al., 2007	Questionnaire and interview	720 AMI patients, 22% female	Delay time, clinical history, pain severity, knowledge of heart attack symptoms	***
Khraim & Carey, 2009	Systematic review	Patients with AMI	Predictors of delay	**
Lovlien et al., 2006	Questionnaire	82 AMI patients, 44 male	Lifestyle, medical history, symptoms, factors influencing delay	**
Løvlien et al., 2007	Questionnaire	533 patients with AMI, 384 male	Reported response, delay time, self-medication	***
McKinley et al., 2009	RCT	3522 people with CHD	ACS Response Index	****
Morgans et al., 2008	Questionnaire	600 emergency department attendees	Coping Responses Inventory, Multidimensional Health Locus of Control, delay time	**
Noureddine et al., 2006	Questionnaire and medical record review	204 acute coronary syndrome (ACS) patients, 72% male	Response to Symptoms Questionnaire, delay time	**
Noureddine, 2009	Secondary analysis of questionnaire data	210 patients with ACS, 70% male	Response to Symptoms Questionnaire	**
Perkins-Porras et al., 2009	Interview and medical note analysis	228 ACS patients, 178 male	History, risk factors, attribution of symptoms, time of admission, symptom onset	**
Riegel et al., 2008	RCT	1777 patients at risk of ACS	Emergency admission, history, pain ACS beliefs	****
Ruston & Clayton, 2007	Interview	44 female patients following cardiac event	Interpretation of symptoms and action taken	**
Sullivan et al., 2009	Questionnaire	796 patients with suspected ischemic heart disease, 77% male	Relationships Scales Questionnaire, intentions about seeking care, Seattle Angina Questionnaire, Perceived risk of MI, depression scale, Beck Anxiety Inventory	***
Thuresson M, 2007	Questionnaire and examination of medical records	1939 ACS patients, 75% male	Interpretation and response to symptoms, patient actions, the decision-making process, delay	***
Tullmann et al., 2007	RCT	115 Over 65 year old AMI patients, 52% female	Response Questionnaire, Control Attitude Scale, Brief Symptom Inventory Anxiety Subscale	***
Turris, 2008	Interview	16 females who sought emergency department care	Experiences of care	**
Turris, 2009	Interview	16 females seeking emergency department care	Reported decision-making strategies and knowledge	**
Turris et al., 2008	Observation and interview	100 hours of observation, interviews with 16 females attending emergency departments, interviews with 3 nurses	Reported interpretation of symptoms and decision-making	**
Vavouranakis et al., 2010	Observation, interview, questionnaire	348 patients with MI, higher ratio males	Delay time, factors for delay	**
Zegrean et al., 2009	Secondary analysis of interview and questionnaire data	135 AMI patients, 72% male	Decision delay, Response to Symptoms Questionnaire, coping strategies	**

### Self-regulatory model of illness behaviour

The most frequently mentioned theoretical framework was the self-regulatory model (SRM) [13] This theory was referred to in nine papers [14-22]. This model proposes that both internal and environmental stimuli influence the behaviour of an individual when faced with a health threat. Internal characteristics include age, gender, ethnicity and environmental stimuli include the influence of significant others. The framework suggests that cognitive and emotional (affective) systems make independent contributions to health and illness behaviour. It outlines three stages of behaviour at the time of a health threat (such as having symptoms of a heart attack). The first stage relates to an individual’s knowledge, attitudes and beliefs regarding the threat which can impact on levels of anxiety and whether a symptom is judged to be serious or not. The second stage refers to how the individual then responds, by formulating an action plan to cope with the symptoms. The final stage describes the evaluation of any action taken by the individual and may include input from significant others.

A randomised controlled trial from the United States of America (US) outlined in three papers reported that the intervention used was based on the self-regulatory model [14-16]. The intervention was designed to address both cognitive and emotional processing of symptoms and to include elements relating to social factors and the development of an action plan. It was delivered by a nurse and included the provision of information relating to symptoms, discussion of emotional responses, role playing scenarios, included family members in sessions and contained the opportunity to develop a personalised “advisory form” to put up at home outlining steps to take at the onset of symptoms. The study found that while the intervention effected change in terms of increased knowledge, attitude and beliefs there was a very limited impact on behaviour. While the intervention group was significantly more likely to take aspirin after symptom onset there was no effect on emergency service use or delay. The authors hypothesised that simple behaviour change (taking an aspirin) was very different from other behaviour change which included psychological barriers (such as calling emergency services or seeking care).

A pilot randomised control trial in older adults with symptoms of heart problems in the US was described as being designed specifically to address the cognitive and emotional elements of the SRM [17]. The intervention was delivered by nurses and consisted of information giving, provision of instructions to keep at home, and discussion of emotional responses using scenarios. This study found a significant increase in knowledge, beliefs (about recognising symptoms and taking action) and perceived control however, there was no difference in attitudes regarding recognising symptoms or getting help. The authors hypothesised that the disparity between participant’s beliefs and their attitudes was due to the belief measurements being related to intended behaviours (if I thought I was having a heart attack I would go to the hospital), however the attitudes questions related more to participants’ perceptions of their own abilities (how sure are you that you could recognise the symptoms of a heart attack).

Structured patient interviews based on the SRM were used in one study [18]. The interviews with women explored mental representations of symptoms, the development of their action plans, and factors influencing evaluation of their action plan. The study found that in addition to socio-demographic factors influencing delay, one of the strongest influences was participants’ perception that they were not likely to have a heart attack. In SRM terms they did not perceive symptoms as a health crisis or threat and thus did not develop a plan of action.

A second qualitative study reported its findings in relation to the SRM [19]. Echoing the study above, this work found that the women interviewed tended not to regard themselves as at risk. The authors hypothesised that the response to symptoms in these participants was more emotionally focussed (coping strategies of denial) rather than action planning (calling emergency services). There was reported ambivalence regarding whether or not to contact medical services, which the authors suggested could be seen as part of the SRM evaluation process. The seventh paper referencing the SRM briefly drew parallels between the finding that people with depression were slower in seeking medical intervention, and the role of cognitive and affective systems in health and illness behaviour [20].

Two papers by the same author drew on the Common-Sense Model of Illness Representation [21,22]. This term appears to mirror work describing the SRM [23]. The study outlined in these papers found that the most frequently reported factor contributing to delay was waiting for symptoms to go away. This waiting behaviour was also associated with having intermittent symptoms and not recognising the symptoms as cardiac.

### Cognitive systems/knowledge regarding the threat

There were a number of papers which while not making direct reference to an underpinning theoretical framework reported data relating to the influence of cognitive systems on health behaviour, a key aspect of the SRM. Studies examined cognitive aspects in particular relating to level of knowledge, symptom expectations, symptom attribution, recognition of symptoms, and perceived seriousness.

One large-scale questionnaire study found an association between limited knowledge of symptoms of a heart attack and late presentation to a hospital [24]. In similar vein another found low knowledge levels in nearly half of patients (46%) [25]. Two papers (one secondary analysis and one questionnaire-based) highlighted that delay could be due to a mismatch between the symptoms being experienced and expectations regarding what cardiac symptoms “should be” [26,27]. Seven further studies (five using questionnaires and two using qualitative interviews) described a failure to interpret pain as originating from the heart and lack of attributing symptoms to a heart attack [28-34]. Women in particular might not recognise symptoms due to their perception of heart attacks as being a male disease [33] and often reported an absence of a uniform pattern of symptoms [34]. Three papers highlighted an association between the perceived severity of symptoms and seeking assistance, with patients and relatives often explaining symptoms as being due to a less serious condition [35-37].

Authors of one qualitative interview study [38] referred to Locker’s work on cue inventories [39]. This theoretical approach suggests that symptoms become organised by an individual into mental lists or records and accounts of typical ways that symptoms manifest themselves. These cue inventories are based on experiences and form part of a stock of knowledge which is drawn on to make sense of a situation. The study found that women had extensive cue inventories which they used to provide continuity between past and present experiences and used as a means of alerting them to signs that were important and required action, and when symptoms were new or had escalated. Early and late hospital attenders could be distinguished by information in their cue inventories. Earlier attenders had mental records of knowledge of symptoms and previous experiences whereas later attenders had knowledge and experience of recent and co-occurring chronic illnesses (suggesting to them that their symptoms were typical or normal).

### Belief systems

The fourth paper from the RCT carried out in the US (based on the SRM) reported that a lower belief score was one of three factors that significantly predicted longer delay time in participants who had received the intervention. The other elements were a higher perceived control and higher anxiety. This paper drew attention to a high level of unexplained variance between patients [40].

### Coping strategies

Seven of the included studies referred to coping strategies or mechanisms used by people experiencing symptoms [32,41-45]. Coping strategies identified included: trying to relax; wishing/praying for symptoms to go; discussing symptoms with others; problem solving; “cognitive avoidance”; resignation; acceptance; positive reappraisal; and self-medication. Kaur et al. [45] discussed the SRM concepts of internal and external coping however they also described a typology developed by Lazarus and Folkman [46] where coping is conceptualised as being either problem-focused or emotion-focused. The authors concluded that interventions should take account of these differing individual coping strategies. The AMI (Acute Myocardial Infarction) Coping Model [47] was described in two papers by the same authors [32,44]. This framework which appears to be closely linked to the SRM model aims to provide an understanding of the impact of emotional responses on behaviour. Greater use of emotion-focused coping was associated with longer care-seeking delay, suggesting that interventions should focus on reducing the use of emotion-focused coping behaviours such as distraction, denial and ignoring symptoms.

Authors of a questionnaire study hypothesised that the psychological strategy of denial may be influential in impeding patients’ ability to make appropriate decisions about seeking medical care [48]. The Safer et al. model of treatment-seeking behaviour [49] underpinned another questionnaire-based study [50]. The authors of this work reported however that this model did not appear to explain why depressed individuals had longer delay times than non-depressed. Gallagher et al. [51] argued that the description of treatment seeking responses as incorporating causal beliefs and coping responses does not sufficiently reflect the complexity of the process described by interview participants. They drew instead on work describing patterns of decision trajectories [52]. This typology distinguishes patients as “knowing and going” (early recognition and treatment-seeking) or “managing an alternative hypothesis” (working through treatments until hypotheses are excluded or there are further symptoms).

### Other theories

#### Attachment theory

One large-scale questionnaire-based study outlined the application of attachment theory to understanding care delay [53]. It found that patient views regarding the trustworthiness of others may be important to address in interventions.

#### Maintaining integrity

Two papers by the same authors [54,55] outlined the social psychological process of maintaining integrity (personal, social and physical) in relation to treatment seeking. The authors described how patients who seek treatment for symptoms suggestive of cardiac disease consider many factors that shape their actions and may strive to make sense of their symptoms and act in ways congruent with maintaining integrity. The women in this interview study reportedly strove to keep intact their image of themselves as good wives, mothers and employees and made efforts to preserve normal daily routines and fulfil role responsibilities. This often resulted in watching and waiting, hoping that symptoms would resolve spontaneously. A later paper by the same author [56] examined the theory of Ways of Knowing [57]. Participants in this study described the risk to others, and (in an echo of the papers above) the effect of treatment-seeking on their social roles and responsibilities. The author concluded that treatment-seeking delay could be a social rather than an individual phenomenon.

#### Social constructionist perspective on gender

One qualitative paper examined how masculinity influences men’s interpretation of chest pain and help-seeking decisions [58]. The authors concluded that that the decision to seek help was a complex process with a number of factors influencing delay including seeking help being viewed as emasculating and symbolically associated with being a hypochondriac or weak. The work highlighted that conceptions of masculinity and reluctance to access healthcare were culturally determined and that interventions needed to take men’s experiences and representations of masculinity into account.

## Discussion

A theoretical approach to review of these papers illuminates the complexity of factors which may contribute to patient delay in seeking professional medical help, and thus provides a useful addition to conventional systematic review methods. The review findings highlight the dominance of the self-regulatory model of illness behaviour in the field. This theory is helpful in focusing the attention of researchers on patient-related factors that may affect the implementation of interventions in addition to scrutinising the system of provision. The approach emphasises a need to examine individual’s subjective experiences of health threats in order to understand the way that they adapt to these threats. The model therefore offers an important mechanism for exploring individual variability in responses to any intervention. It emphasises that people are active problem-solvers who select and manage threats and therefore provides insight regarding why the onset of chest pain may not immediately trigger help-seeking behaviour in all patients.

The review highlights that a differing perception of seriousness may underlie whether action is taken or not, with included studies describing a failure to attribute symptoms to heart problems. The important role of coping procedures in the process was also highlighted, with cognitive and behavioural actions to manage health threats delaying help seeking. The review suggests that understanding and addressing these coping behaviours could be of key importance in developing successful interventions. The work emphasises the challenge in changing behaviour via information-based interventions, with a distinction drawn between simple behaviour change and complex behaviour change including psychological barriers. While there was some evidence of low knowledge levels interventions increasing knowledge did not necessarily lead to changed action, with both cognitive and emotional aspects also influencing behaviour.

While providing important insights, the SRM is only one of a considerable range of social cognition models concerned with individuals’ causal explanations of health-related events [59]. The model has conceptual similarities with the Health Belief Model and social learning theory, however it differs in terms of its timelines, causes and coping procedures [13,60,61]. The weak empirical evidence underpinning the SR model has been highlighted, with criticism that it offers little guidance on the design of interventions [59,62]. It may be important to note that while the review found a paucity of evidence available from intervention studies, the randomised controlled trial in this review based on the SRM demonstrated no change in help-seeking behaviour.

The review suggests that effective interventions need to be multi-faceted and address variance in patient perceptions of threat. Interventions should identify and explore patient coping strategies, and include not just information-provision but provide individuals with opportunities to relate information to themselves and their individual circumstances and plan how personal barriers to them seeking help may be prepared for and overcome. Attention to individualising design of interventions is further suggested by the review highlighting gender variation in coping behaviours and responses. This creates challenges for large-scale publicity campaigns or information provision however reinforces that multiple methods of tackling the issue are needed.

While it is argued that the theoretical frameworks such as the SRM takes account of individual views of their environments as well as of their selves [62] the papers included in this review which referred to other theoretical perspectives placed more emphasis on the important influence of patients’ social roles and responsibilities in decision-making regarding seeking treatment. This aspect seemed not to have been identified in the SRM studies. In particular the work which described women’s perceptions of their role which created obstacles to early help seeking may be important in understanding gender differences in response to symptoms. Also, the work which reported the influence of perceptions of masculinity suggested that interventions should be differentiated according to the gender of participants. One further paper suggested that patient’s views regarding the trustworthiness of services may be influential. The dominance of the SR model in the field may not be recognising the full range of potentially influential factors. Several contextual factors such as socioeconomic position, health literacy, and systemic discrimination/cultural safety are not considered within the theories which were reported. These elements are known to have an important impact on patient’s access to health services and may thus be key areas of omission in interventions developed from SRM frameworks.

This review found a predominance of work using questionnaire and qualitative study designs, with only a single RCT reported in several papers. By adopting a broad study design inclusion criterion we were able to draw upon a range of studies that would typically be excluded in a systematic review to inform our findings. By adopting a theory-based approach to further analyse the papers we argue that this review has been able to make a valuable contribution to knowledge in the field. The inclusion of studies across a range of designs however presents challenges in terms of quality appraisal which we addressed by designing a grading system which could be used across all study types. However, this may be viewed as controversial and criticised as being overly simplistic. Many tools for critically appraising papers exist in order to grade studies within each design with few generic tools available that are applicable to health research [10]. In the search for new forms of review methodology it may be timely to examine alternative forms of quality assessment. Reviews which seek to identify and synthesise data relating to theoretical underpinnings may perhaps be best served by a tool which evaluates the quality of the theoretical lens. Checklists for qualitative studies may include an item relating to theoretical approach however this tends not to be the case for quantitative designs. This may perpetuate a perception that there is less need for the theoretical rationale to be reported in quantitative studies and may have been influential in this review predominantly including questionnaire-based and qualitative interview studies.

The work reported here highlights however that the theoretical underpinnings of a study are of importance for all authors to recognise and report if assumptions underpinning how and why an intervention may work is to be fully understood. Theory-based approaches have been described as filling an “evaluation deficit” by identifying assumptions and tacit understandings that implementers may have regarding how a programme should work [62,63]. It is argued that these assumptions need to be brought to light in order to describe the steps to be taken in implementation of a programme and the mechanisms that need to happen [64]. This theory-based review has echoed the argument made by proponents of realistic evaluation that interventions need to pay attention to people embedded in their context (such as by examining coping mechanisms and perceptions of role and responsibilities) as it is individuals who change rather than programmes that make things change [5]. There is perhaps a need for researchers to more explicitly underpin study design and evaluation with a theoretical rationale, and for this theoretical rationale to be clearly communicated in scholarly publications.

This work used the set of papers identified by the prior review which had used the cut-off of 2006 due to a significant change in patient care following the introduction of percutaneous coronary interventions. While this rationale for the review of interventions may be justifiable, we accept that theory-based work outside this time frame may have been advantageous to explore and include. The study described here was carried out as exploratory work to investigate whether an alternative method of analysis and synthesis could yield additional insights into the data from a set of papers. Having tested and confirmed the potential value of this approach we would recommend that date inclusion criteria should relate to theoretical justification rather than intervention type in future work. A further date-related limitation of the current study may be that the searches were completed in 2011, with potentially further relevant papers published more recently.

## Conclusions

The extension of systematic review methods to include the examination of theoretical underpinnings yields additional insights into complex interventions. The process of decision-making regarding whether to seek medical assistance following the onset of chest pain that could be due to a heart attack is multi-factorial requiring multi-faceted complex interventions. Important elements to address in any intervention seem to be the perceived seriousness of the symptoms, the process of coping with the symptoms adopted, and the perceived impact on role and responsibilities. These elements require further evaluation via intervention studies.

## Competing interests

The authors declare that they have no competing interests.

## Authors’ contribution

SB conceived and designed the study, carried out the synthesis and drafted the manuscript. PA contributed to the conception, acquisition of data and read and commented on the manuscript. Both authors read and approved the final manuscript.

## Pre-publication history

The pre-publication history for this paper can be accessed here:

http://www.biomedcentral.com/1471-2288/13/15/prepub
